# *Trichomonas vaginalis* infection and the diagnostic significance of detection tests among Ghanaian outpatients

**DOI:** 10.1186/s12905-018-0699-5

**Published:** 2018-12-27

**Authors:** Richard Harry Asmah, Rita Ofosuaa Agyeman, Noah Obeng-Nkrumah, Harriet Blankson, Georgina Awuah-Mensah, Momodou Cham, Listowell Asare, Patrick Ferdinand Ayeh-Kumi

**Affiliations:** 1 Department of Medical Laboratory Sciences, School of Biomedical and Allied Health Sciences, University of Ghana, Korle-bu, Accra, Ghana; 2 School of Life Sciences, University of Nottingham, Nottingham, UK; 3 Camboni Catholic Hospital, Sogakope, Ghana

**Keywords:** Ghana, Trichomoniasis, Wet mount, Polymerase chain reaction, Risk factors

## Abstract

**Background:**

There is little data on *Trichomonas vaginalis* infection in Ghana. This study evaluated the prevalence of trichomoniasis using different diagnostic methods and determined the risk factors for infection in patients.

**Methods:**

A structured questionnaire was administered. Vaginal swabs, urethral swabs and urine specimens were obtained from consenting patients; and the samples processed following standard protocols. The presence of *T. vaginalis* was determined using wet mount microscopy and polymerase chain reaction (PCR) as gold standard. We also assessed the diagnostic performance the JD’s *Trichomonas* V® rapid antigen test to inform clinical practice.

**Results:**

The PCR assay detected *T. vaginalis* positivity in 64 of 150 patients (42.6, 95%CI:35.0, 50.6) including all positive samples of wet mount microscopy and JD’s *Trichomonas* V® test. Wet mount microscopy showed low sensitivity (31.6%), high specificity (100%), moderate positive predictive value (75.0%), moderate positive likelihood ratio (3.0), and weak agreement (Cohen’s kappa, 0.283) with PCR assay. The JD’s *Trichomonas* V® test displayed lower sensitivity (25.0%), specificity (83.3%), and weaker measure of agreement (Cohen’s kappa, 0.233) with PCR. In multivariate analysis, the strongest independent predictor for *T. vaginalis* was female gender [adjusted odds ratio (AOR), 24.89; 95% confidence interval (CI): 10.58, 51.21; *P*-value< 0.001]. Knowledge of STI showed a protective effect against infection with the parasite (AOR, 0.13; 95%CI: 0.07, 0.29; *P*-value< 0.017).

**Conclusion:**

The sensitivity of wet mount microscopy was low for *T. vaginalis* screening in our region. The JD’s *Trichomonas* V® test should not be considered as an alternative test. We recommend mandatory PCR assay for confirmation of negative wet mount results.

## Background

Trichomoniasis is a common sexually transmitted infection (STI) caused by the protozoan parasite *Trichomonas vaginalis*. The highest prevalence of trichomoniasis has been reported from resource-limited countries and among deprived populations in developed countries [1, 2]. Of the estimated 153 million cases of trichomoniasis, 25.64% are from the African [1, 3]. Trichomoniasis is mainly transmitted by sexual intercourse with an infected person and affects the urogenital system, vagina, and urethra [4, 5]. The clinical presentations of the infection in women include yellowish-green frothy vaginal discharge with vulvar irritation which may be confused with bacterial vaginitis, whereas infected men may briefly have urethral irritation, mild discharge, or slight burning after urination or ejaculation [3, 6–9]. Factors such as low level of education, poor hygiene, low socio-economic status, and multiple sexual partners have been associated with high prevalence of trichomoniasis [10, 11].

There is paucity of data on *T. vaginalis* infection in Ghana [12, 13]. The few available studies were conducted among women. And most of these studies were performed with wet mount microscopy. Wet mount microscopy is reported to have low sensitivity of 45–60% compared with polymerase chain reaction (PCR) tests [14, 15]. Consequently, the occurrence of trichomoniasis and its associated public health implications in Ghana may be underestimated. Other methods such as culture are recommended but it takes a long time for diagnosis and still requires expert microscopy. Simple-to-perform rapid diagnostic tests are emerging globally as the preferred point-of-care diagnosis for protozoan infections albeit with varying sensitivities compared to wet-mount. The JD’s *Trichomonas* V® antigen-based rapid diagnostic strip test (Biotech™ Corp., China) that was recently introduced in our district but with no data on its reliability. To inform practice, we assessed the diagnostic performance of JD’s *Trichomonas* V® test for *T. vaginalis* infections, compared to wet mount microscopy, and using PCR as the gold standard. We also report on the associated risk factors for trichomoniasis in our setting.

## Methods

### Study settings

This study was conducted at the Comboni Hospital in the South Tongu District of Ghana. The District has an estimated population of 86,000 with an annual growth rate of 2.5% [16]. The hospital has an annual outpatient attendance of 47,528. It has a bed capacity of 50 with 8 doctors. The Comboni hospital has a laboratory that provides some microbiological services, including routine parasitological examinations, but does not perform bacterial culture and antibiotic sensitivity testing. The laboratory offers immunology, biochemistry, as well as haematology and transfusion services. The hospital receives referral cases from other health centres in the district.

### Study design

We conducted a hospital-based prospective cross-sectional study from May to June 2014. Sampling involved two steps: (i) recruiting patients and interviewing them; and (ii) collecting study samples for laboratory investigations. The study participants comprised patients referred to the Comboni hospital for laboratory investigation of any type of sexually transmitted diseases (STD) whether or not they were sexually active. From this cohort, we investigated patients for *T. vaginalis* infections. Prior to the commencement of work, the research team conducted meetings with the Comboni hospital authorities, staff, and patients to explain the objectives of our study including benefits, potential risks and discomforts. Patients were recruited based on willingness to fully participate and provision of written consent. Informed written consent were provided by patients before enrollment into the study. For patients aged < 18 years, we sought for informed consent from parents or legal guardians as well as verbal consent by the patient to willingly partake in the study. Patients or legal guardians who provided informed consent also permitted that the data to be collected could be published in a peer-reviewed journal. A structured questionnaire (Additional file 1) was administered through interview to collect information on demographics, knowledge, attitudes and patients’ practices that predispose them to *T. vaginalis* infections. Because patients were recruited at the laboratory, we conducted a physician-assisted work through of participants’ hospital folders to ascertain signs and symptoms, confirm diagnosis and record treatment history. Patients on past (within 2 weeks) or current treatment for suspected sexually transmitted infections were excluded from study. Approval for the study (ID: MS-Et/M.5-P.3.3/2013–2014) was granted by the Ethical and Protocol Review Committee, University of Ghana Medical School, Korle-Bu.

### Sample collection and processing

The primary outcome of the study was the detection of *T. vaginalis* in patients coming for STD checkup. Specimen collection and investigations were conducted following standard protocols but independent of the routine standard operative procedures for laboratory diagnosis of STD at the Comboni hospital. In our study, vaginal swabs were requested from females. Males provided urethral swabs. At the same time, about 15–20 mL of first catch urine samples were collected from both female and male participants. Swab specimen from each patient were agitated vigorously in 1 ml of physiological saline (0.85%). The urine samples were centrifuged at 1000 rpm for 5 min and decanted to ensure the viability of trichomonads for wet mount microscopy. All patients who provided samples and accompanying data were de-identified and allotted arbitrary numbers to ensure anonymity. Patients found infected with parasites as well as those found with ailments not targeted by the project were referred to hospital for treatment.

#### Wet mount microscopy

Aliquot of the swab elute and decanted urine samples per patient were subjected to wet mount microscopy within 20 min after specimen collection. They were examined for motile trichomonads using × 100 and × 400 magnification. Three slides were prepared for each specimen to increase chances of recovering *T. vaginalis. Trichomonas vaginalis* was recorded as present if ≥1motile trichomonads were seen at any magnification.

#### Rapid antigen test

The JD *Trichomonas vaginalis* rapid tests were performed according to manufacturer’s (Biotech™ Corp., China). Briefly, 0.5 mL of the swab elute was mixed in 0.5 mL of test buffer (0.01% Tris-Hcl and 0.05% NaN3, PH 7.5) for 10 s. The JD’s *Trichomonas* V® test strip was placed in each mixed test buffer, and the result read visually after 15 min. For urine sediments, 0.5 ml of sample buffer was added to 0.5 mL of the sediments and mixed well for about 10 s. For each urine-buffer test buffer, a JD’s *Trichomonas* V® test strip test strip was placed in the mixture, and the result read visually after 15 min.

### Polymerase chain reaction tests

Aliquots of swab elutes and urine specimens from each patient were separately placed in DNA guard (Biometrica, Co. USA) until further work-up. Polymerase chain reaction (PCR) targeting the beta-tubulin genes of *T. vaginalis* was used for the detection of the organism. The targeted genes encode the amino acid sequence of beta-tubulin protein, a major component of the *T. vaginalis* flagella and cytoskeleton [17]. The Qiagen DNA extraction kit (column method) was used to extract DNA. The primer set used was BTUB 9/2 [18] which targeted a conserved region of the beta-tubulin genes *btub1, − 2, and − 3* (GenBank accession numbers: *btub1*, L05468; *btub2*, L05469; and *btub3*, L05470) of *T. vaginalis.* The primer set was designed to amplify a DNA product of 112 bp from the three beta-tubulin genes. We used the sequence of forward primer (BTUB 9, 5’ CAT TGA TAA CGA AGC TCTTTA CGA T 3: positions 850 to 874) and reverse primer (BTUB 2, 5’ GCA TGT TGT GCC GGA CAT AAC CAT 3′: positions 961 to 938). A final volume of 25 μl was used for PCR amplification reaction. This contained 10 μl of DNA template, 0.625 μl of each primer, 0.5 μl of each of the four dNTPs (Sigma, Co. USA), 0.125 μl of Taq polymerase (Biopioneers, Co. USA), 5 μl of PCR buffer (Biopioneers, Co. USA) and 6.625 μl nuclease-free water. The following PCR conditions were used: an initial denaturing step of one minute at 95 °C followed by 40 cycles of denaturation at 95 °C for 45 s, annealing at 52 °C for 45 s and extension at 72 °C for 1 min and then a final extension at 72 °C for 2 min. The PCR products were run on 2% agarose gel with ethidium bromide and visualized in visualized under ultraviolet trans-illuminator (JL Berker, Germany).

### Data analysis

Data were entered into a Microsoft Excel spreadsheet and summarized using graphs and tables. Statistical analyses were performed using Epi info™7 statistical software package (CDC, Atlanta. The definitive consideration for *T. vaginalis* infection was positivity by PCR in any of two included patient specimens. Discrepant specimens (those with different results outcome for vaginal/urethral swab negative and urine samples) were determined to be *T. vaginalis* positive by performing PCR. The gold standard was PCR positivity. The diagnostic performance of detection methods was calculated by using the defined gold standard with confidence intervals based on normal approximation to binomial distribution. Agreement measures between detection methods was determined with Cohen’s kappa tests. For descriptive statistics we calculated percentages with 95% confidence interval (95% CI), mean ± standard deviation (SD). Univariate comparisons were computed with Chi-square tests and unadjusted Odds ratios (OR) at 95% confidence interval (CI). In multivariate analysis, logistic regression was conducted with adjusted odds ratios (AOR) using all variables that had *p* < 0.5 in the univariate comparisons. Point estimates of statistical significance were indicated with 2 tailed *P*-values < 0.05. Association between age and *T. vaginalis* was demonstrated with Locally Weighted Scatterplot Smoothing (LOESS) was generated using Tricube kernel smooth fit line with 60% of points to fit.

## Results

Overall, 203 patients were eligible for inclusion. Of these, 150 provided informed consent and were enrolled in the study. There were 110 females and 40 male patients with a mean age of 28.67 years (ranging from 16 to 55 years). Out of this, 110 (73.33%) were females with a mean age of 27.81 years (ranging from 16 to 50 years), and 40 (26.67%) were males with a mean age of 31.05 years (ranging from 20 to 55 years). All included participants were sexually active. Among the female subjects, 45 (40.91%) were pregnant. Genitourinary symptoms were observed in 81 of 150 patients (females,71; male, 10). The commonest symptoms associated with the included cases was genital discharge (70.4%, *n* = 57/81, females, 54; males, 3), followed by itching in 61.7% (females, 49; males, 1) of the cases and painful micturition (females, 18; males, 8) in 32.1 of cases.

### Occurrence of *T. vaginalis*

Table 1 shows the occurrence of *T. vaginalis* from patients’ samples*.* A total of 300 samples (females: 110 vaginal swabs plus 110 urine samples; males: 40 urethral swabs and 40 urine samples) were received from study participants. Of the 300 samples tested, 127 were positive by at least one of the techniques used. None of the techniques could detect all positive samples. The PCR assay detected *T. vaginalis* positivity in 64 of 150 patients (42.6%; 95%CI:35.0, 50.6), and this was more prevalent in females (*n* = 53/110, 48.1%; 95%CI:39.1,57.2) than in males (*n* = 11/40, 27.5%; 95%CI: 16.1,42.8). The PCR assay was positive for 10 of 30 males and negative for all females (*n* = 0/30) without genitourinary symptoms. Of the 150 subjects studied, 24 (16.0%; 95%CI:10.9, 22.7) were positive for *T. vaginalis* with wet mount microscopy. When data was stratified across gender, 24 of the 110 female subjects (21.82, 95% CI 14.51, 30.70) were positive for *T. vaginalis* compared to none for the male patients. The wet mount microscopy was positive for 4 of 39 females and negative for all males (*n* = 0/30) without genitourinary symptoms. The JD’s *Trichomonas* V® test detected *T. vaginalis* in 19 (12.6%; 95%CI: 8.3, 18.9) of 150 participants— 15 (13.6%; 95%CI: 8.4, 21.9) in 110 female patients and 4 (10.0%; 95%CI: 3.9,23.1) in 40 male patients. The rapid test was positive for 4 of 30 males and negative for all females (*n* = 0/39) without genitourinary symptoms.

**Table 1:** Detection of *Trichomonas vaginalis* in patients with Wet Mount Microscopy, JD’s Trichomonas V® Rapid Antigen Test and PCR

Patients and specimens	Diagnostic methods					
	Wet Mount Microscopy		Rapid Antigen Test		PCR	
	No.	No. positive (%) [95%Cl^a^]	No.	No. positive (%) [95%Cl]	No.	No. positive (%) [95%Cl]
All patients	150	24 (16.0) [10.9, 22.7]	150	19(12.6) [8.3, 18.9]	150	64(42.6) [35.0, 50.6]
Gender						
Females	110	24 (21.8) [15.1, 30.4]	110	15 (13.6) [8.4, 21.9]	110	53(48.1) [39.1, 57.2]
Males	40	0	40	4 (10.0) [3.9, 23.1]	40	11 (27.5) [16.1, 42.8]
All specimens	300	25 (8.3) [5.7, 12.1]	300	28 (9.3) [6.5, 13.2]	300	127 (42.3) [36.8, 47.9]
Specimen type						
Vaginal swab	110	23 (20.9) [13.7, 29.7]	110	15 (13.6) [8.4, 21.9]	110	53 (48.1) [39.1, 57.2]
Female urine	110	2 (1.82) [0.5, 6.4]	110	9 (8.1) [4.3, 14.8]	110	52 (47.3) [38.2, 56.5]
Urethral swab	40	0	40	4 (10.0) [3.9, 23.1]	40	11 (27.5) [16.1, 42.8]
Male urine	40	0	40	0	40	11 (27.5) [16.1, 42.8]
Without genitourinary symptoms						
Females	39	4 (10.3) [4.1, 23.6]	39	0	39	0
Males	30	0	30	4(13.3) [5.3, 29.8]	30	10 (33.3) [19.2–51.2]
With genitourinary symptoms						
Females	71	20 (28.1) [19.0, 39.5]	71	15 (12.1) [13.3, 33.7]	71	53 (74.6) [63.5, 83.2]
Males	10	0	10	0	10	0
Genital discharge						
Females	54	14 (21.1) [16.1, 38.9]	54	14 (21.1) [16.1, 38.9]	54	38(70.3) [157.1, 80.6]
Males	3	0	3	0	3	0
Itching around genital area						
Females	49	11 (22.4) [13.1, 35.8]	49	14 (28.5) [17.9, 42.4]	49	35 (71.4) [57.6, 82.9]
Males	1	0	1	0	1	0
Painful micturition						
Females	18	8 (44.4) [25.6, 66.3]	18	10 (55.5) [33.7, 75.4]	18	13 (72.2) [49.1, 87.5]
Males	8	0	8	0	8	0
Genital ulcers						
Females	5	4 (80.0) [37.6, 96.4]	5	5 (100.0) [56.5, 100.0]	5	5 (100.0) [56.5, 100.0]
Males	1	0	1	0	1	0
Burns						
Females	0	0	0	0	0	0
Males	2	0	2	0	2	0

^a^Clinical diagnosis, physician-assisted clinical summary/diagnosis; *CI* confidence interval, *VS* high vaginal swab, *UTI* urinary tract infection, *STI* sexually transmitted infection, *PID* pelvic inflammatory disease, *PCR* Polymerase Chain Reaction; others, clinical diagnosis not related to genitourinary diseases and those without specific diagnosis

### Sample type and test positivity

We compared tests positivity of vaginal/urethral swabs and urine by wet mount microscopy, the JD’s *Trichomonas* V® test and PCR assay. The PCR detected could detect all positive samples of wet mount microscopy and the JD’s *Trichomonas* V® test. Of the 53 female patients with vaginal swabs positive for *T. vaginalis* by PCR, 52 also had urine samples positive for the parasite by the same technique. Similarly, all 11 male cases with urethral swab positive for *T. vaginalis* by PCR also had positive urine specimens for the parasite. For wet mount microscopy, 24 *T. vaginalis* cases were positive for 23 of 110 vaginal swabs compared to 2 of 110 urine samples. Only 1 patient had discrepant specimen (negative vaginal swab with positive urine sample). Both samples were determined by PCR to be positive for *T. vaginalis*. The JD’s *Trichomonas* V® test had 6 female and 4 male patients with discrepant results. The test identified *T. vaginalis* in 15 of 110 vaginal swabs and 9 of corresponding urine samples. It also detected *T. vaginalis* in 4 of 40 urethral swabs but all the corresponding urine swabs were negative for the parasite. The discrepant samples were all *T. vaginalis* positive by PCR.

### Performance of diagnostic techniques

The diagnostic performance of wet mount microscopy and the JD’s *Trichomonas* V® test were compared using results of the PCR assay as gold standard (Table 2). Wet mount microscopy showed low sensitivity (31.6%) but high specificity (100%) with moderate to high positive predictive values (75.0%) and positive likelihood ratio (3.0) for all patients. The microscopy recorded its best sensitivity score among asymptomatic (40.0%) and female (39.2%) patients. The least sensitivity (0%) was noted for male patients. In comparison, there was weak agreement (Cohen’s kappa, 0.283) between wet mount microscopy and PCR assay. The kappa improved to 0.533 only when asymptomatic cases were considered. Across all patients, the JD’s *Trichomonas* V® test displayed lower sensitivity (25.0%) and specificity (83.3%) compared to wet mount microscopy but with better positive predictive values (83.3%) and positive likelihood ratios (5.0). A weak agreement (Cohen’s kappa, 0.233) was noted between the rapid antigen test and PCR assay— and this was evident across all patient groups. Interestingly, although wet mount microscopy and the JD’s *Trichomonas* V® test had moderate to high specificities, their agreement was moderate (Cohen’s kappa, 0.541).

**Table 2:** Diagnostic performance of Mount Microscopy and JD’s Trichomonas V® Rapid Antigen Assay compared to PCR in the detection of *T. vaginalis* among females

Diagnostics^a^	PCR							
	Test^a^	Percent sensitivity (95%CI)	Percent Specificity (95%CI)	Predictive values		Likelihood ratio		Cohen’s Kappa values (95%CI)
	Pos.; Neg.			Positive (95%CI)	Negative (95%CI)	Positive	Negative	
Wet Mount Microscopy	18/24; 87/126	31.6 (20.3, 45.3)	93.5 (85.9, 97.3)	75.0 (52.9, 89.4)	69.1 (60.1, 52.5)	3.0 (1.4, 6.2)	0.45 (0.34, 0.59)	0.283(70.0%) (0.15, 0.42)
Females	18/24; 58/86	39.2 (25.4, 54.6)	90.6 (80.0, 96.1)	75.0 (52.9, 89.3)	67.4 (56.3, 76.9)	3 (1.4, 6.2)	0.48 (35.2, 66.1)	0.313 (0.151, 0.482)
Males	0/0; 29/40	0	100 (85.4, 100)	–	72.5 (55.8, 84.8)	–	0.37 (0.23–0.64)	0.00
Symptomatic patients	14/20; 28/61	33.3 (20.0, 49.6)	86.7 (72.5, 94.4)	70.0 (45.6, 87.1)	54.1 (40.9, 66.7)	2.3 (1.1, 4.8)	0.85 (62.8, 1.1)	0.109 (−0.057, 0.276)
Asymptomatic patients	4/4; 59/65	40.0 (13.6, 72.6)	100 (92.3, 100)	100 (39.5, 100)	0.9 (80.3, 96.1)	–	0.10 (0.05–21.8)	0.533 (0.217,0.849)
Antigen Test	15/19; 87/132	25.0 (15.1, 38.1)	96.7 (89.8, 99.1)	83.3 (57.7, 95.5)	65.9 (57.1, 73.7)	5.0 (1.7, 14.3)	0.52 (0.41, 0.66)	0.233 (0.103, 0.364)
Females	13/15;57/95	25.4 (14.73, 9.9)	96.6 (87.2, 99.7)	86.6 (58.3,97.6)	0.6 (49.4, 69.7)	6.5 (1.78, 23.9)	0.67 (0.51, 86.4)	0.232 (0.096, 0.368)
Males	3/4; 29/36	0.3 (0.8, 64.3)	96.7 (80.9, 99.8)	75.0 (21.9, 98.6)	80.5 (63.4, 91.2)	3 (0.51, 17.9)	0.24 (0.12, 0.48)	0.333 (−0.001, 0.667)
Asymptomatic patients	3/4; 55/65	23.1 (6.1, 54.0)	98.2 (89.1, 99.9)	75.0 (21.9, 98.6)	84.6 (73.1, 91.9)	3 (0.50, 0.18)	0.18 (0.10, 32.3)	0.290 (0.004, 0.576)
Symptomatic patients	13/15; 28/66	25.4 (14.7, 39.9)	93.3 (76.4, 98.8)	86.6 (58.3, 97.6)	42.4 (30.5, 55.1)	6.5 (1.7, 23.9)	1.4 (1.1, 1.7)	0.151 (0.024, 0.278)

^a^Pos. (a/b), true positives as confirmed with PCR/total positives from diagnostic test; Neg. (c/d), true negatives as confirmed with PCR/total negatives from diagnostic test; *CI* confidence interval, *PCR* polymerase chain reaction

### Univariate comparisons

We compared patients’ characteristics and their odds ratios (OR) by univariate analysis for potential risk factors of *T. vaginalis* infection. *Trichomonas vaginalis* infection was defined as test positivity to PCR assay for any of included patient’s sample. The unadjusted odds ratio of having a trichomonas infection was 2.45 (95% CI, 1.11, 5.39; *P*-value = 0.026) when gender was female (Table 3). Being a minor, young adult or middle-aged adult was not associated with the occurrence of *T. vaginalis* infection. Regarding marital status, no significant association was observed between *T. vaginalis* infection and being married, divorced, or widowed. Rather, single patients were less likely to have *T. vaginalis* infection (OR,0.45; 95%CI:0.23, 0.87; *P*-value = 0.021). The unadjusted odds ratio of having a trichomonas infection was 3.23 (95% CI, 1.23, 8.48; *P*-value = 0.019) for uneducated subjects compared to those with any form of education. The following patient’s characteristics were associated with a reduced likelihood of *T. vaginalis* infection: employment (OR, 0.14; 95%CI:0.07, 0.30; *P*-value< 0.001), pregnancy (OR, 0.37; 95%CI: 0.17, 0.70; *P*-value = 0.011). When data was analyzed using sexual health behaviours (Table 4), participants with knowledge of STI (OR, 0.08; 95%CI:0.04, 0.19; *P*-value< 0.001) or whose partners had no other sexual partners (OR, 0.09; 95%CI:0.05, 0.31; *P*-value< 0.001) had significantly reduced odds of *T. vaginalis* infection. Similarly, subjects with no previous STI record (OR, 0.0.032; 95%CI:0.01, 0.09; *P*-value< 0.001) or who had no sexual partners in the past 1 year (OR, 0.32; 95%CI:0.13, 0.71; *P*-value< 0.001) or were less likely to have the parasite.

**Table 3:** Univariate analysis of patient’s characteristics for potential risk factors of *T. vaginalis* infection

Covariates^a^	Patients (*n* = 150)	Patients with *T. vaginalis*		Unadjusted Odds ratio	95%CI	*P*-value
		Yes (*n* = 64)	No (*n* = 86)			
Demographics						
Female gender	110	53	57	2.45	1.11, 5.39	0.026
Age (±SD)^b^		31.12 ± 12.12	19.35 ± 7.89	2.03	1.06,3.92	0.001
Age groups (years)						
< 18 (minors)	10	6	4	2.12	0.57,7.85	0.326
18–35 (young adults)	84	33	51	0.73	0.38,1.40	0.406
35–55 (middle-aged adults)	56	25	31	1.14	0.58,2.18	0.735
Locality						
Inner town	85	33	52	0.69	0.36, 1.33	0.318
Town outskirt	33	19	14	2.17	0.99,4.75	0.072
Village	32	12	20	0.76	0.34,1.69	0.551
Marital status						
Single	42	24	29	0.45	0.23, 0.87	0.021
Married	63	22	33	0.84	0.43, 1.65	0.732
Divorced widowed	23	11	12	1.57	0.65, 3.83	0.363
Pregnant	45	12	33	0.371	0.17, 0.79	0.011
Education						
None	22	15	7	3.23	1.23, 8.48	0.019
Basic school	59	20	39	0.55	0.27, 1.07	0.093
S.H.S	37	13	24	0.66	0.31, 1.42	0.340
Tertiary	32	16	16	1.40	0.64, 3.06	0.427
Employed	85	20	65	0.143	0.07, 0.30	< 0.001

^a^*T. vaginitis* infection was defined with positivity to PCR diagnostic tests; *SD* standard deviation, *CI* confidence interval, *S.H.S* Senior High School, *STI* sexually transmitted disease

^b^Age was considered as a continuous variable from 16 to 55 years

**Table 4:** Univariate analysis of patients sexual and health characteristics for potential risk factors of *T. vaginalis* infection

Covariates^a^	Patients (*n* = 150)	Patients with *T. vaginalis*		Unadjusted Odds ratio	95%CI	*P*-value
		Yes (*n* = 64)	No (*n* = 86)			
Knowledge of STI	96	22	74	0.08	0.04, 0.19	< 0.001
Previously had STI						
No	103	22	81	0.032	0.01, 0.09	< 0.001
Yes	22	10	12	1.36	0.54, 3.41	0.634
No idea	25	12	13	1.33	0.56, 3.14	0.658
Showed symptoms of STI	81	20	61	0.17	0.08, 0.35	< 0.001
Genital discharge	57	38	19	5.15	2.52,10.51	< 0.001
Itching around genital area	50	35	15	5.71	2.71, 12.01	< 0.001
Painful micturition	26	13	13	1.43	0.61, 3.34	0.513
Genital ulcers	6	5	1	7.20	0.82, 63.25	0.083
Urethral burns	2	0	2	0	–	0.507
No. of sexual partners in past 1 year						
0	38	9	29	0.32	0.13, 0.71	0.007
1	112	55	57	3.11	1.35, 7.16	0.007
Partner(s) had other sexual partner(s) in past 1 year						
No	89	20	69	0.09	0.05, 2.13	< 0.001
Yes	28	15	13	1.72	0.75, 3.93	0.210
No idea	23	13	10	1.93	0.79, 4.75	0.172
No condom use	101	50	51	2.15	1.17, 5.09	0.021
Alcohol use	44	9	35	0.87	0.344, 2.22	0.7775
Co-infection with yeast	79	19	60	1.65	0.56, 4.89	0.3686

^a^*T. vaginitis* infection was defined with positivity to PCR diagnostic tests; *CI* confidence interval, *STI* sexually transmitted disease

### Multivariate risk factor analysis

**Fig. 1:**
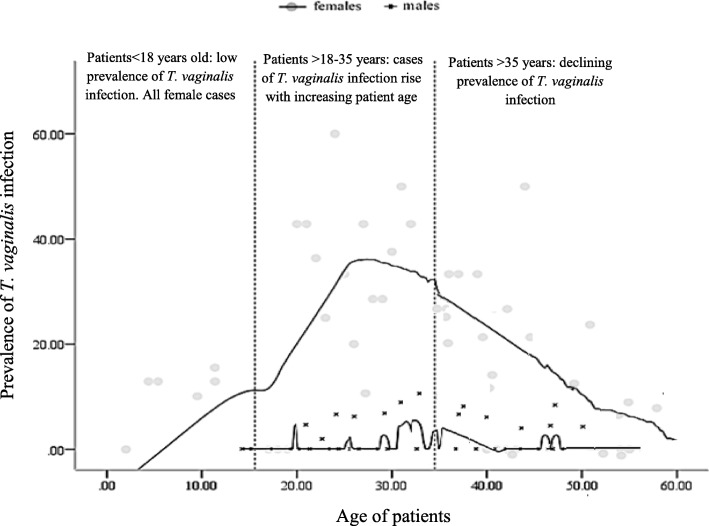
Profile of *Trichomonas vaginalis* prevalence across patients’ age with Tricube Kernel smooth fit line generated with local polynomial regression at 60% of points to fit. Over the period of study, *T. vaginalis* cases were predominant in females. There is apparent relationship between age and infection prevalence among females that peaks at approximately age 26 years

Overall 14 of 40 risk factor sub-levels included in the univariate analysis were significantly associated with *T. vaginalis* infection at the *p* < 0.05 level (Table 4). The results of the multivariate analysis are presented in Table 5. After adjustment for all 14 factors in the in the final multivariate model, 3 variables were significantly associated with *T. vaginalis* infection. The strongest independent predictor for infection was the female gender (AOR, 24.89; 95%CI: 10.58, 51.21; *P*-value< 0.001). Knowledge of STI showed a protective effect against *T. vaginalis* infection. Patients with knowledge of STI had a significantly reduced risk of infection (AOR, 0.13; 95%CI: 0.07, 0.29; *P*-value< 0.017). Age was also significant risk factor for *T. vaginalis* infection. The multivariate analysis show that with every 1 year increase in age doubled the risk of infection with *T. vaginalis* (AOR = 1.93, 95%CI: 1.11, 3.90, *p* = 0.011). The relationship between *T. vaginalis* infection and age was best described with a LOESS Tricube Kernel smooth fit line with local polynomial regression at 60% (Fig. 1). The correlation between *T. vaginalis* and subject’s age was more apparent in females and less dominant among the elderly.

**Table 5:** Multivariate analysis for risk factors of *T. vaginalis* infection

Risk factor^a^	Level	Adjusted Odds Ratio	95%CI	*P*-value
Female gender	Yes/No	24.87	10.55, 51.19	0.001
Age (±SD)	1 year increase	1.41	1.09, 3.54	0.013
Knowledge of STI	Yes/No	0.16	0.08, 0.31	0.014

^a^The predictive accuracy of the models evaluated by Hosmer and Lemeshow goodness-of-fit test was non-significant with *P*-value > 0.05 suggesting that the model predicted accurately on average. The discriminatory power of the multiple logistic regression analysis as measured by the area under the ROC curve was 0.791. Stepwise modelling was adjusted for univariate variables with *P*-value < 0.05

## Discussion

Routine attempts towards the detection and treatment of Trichomoniasis has gained prominence owing to its recent association with HIV, adverse gynaecological and obstetric outcomes and infertility [19–22]. *Trichomonas* infection attracts little attention in sub-Sahara Africa perhaps because most infected people do not show any symptoms and when left untreated serve as asymptomatic carriers [22, 23]. Poor diagnostic techniques for *T. vaginalis* also compounds the burden.

In this study, 150 patients visiting the laboratory for STI check-up were examined for *T. vaginalis* and among these, 64 (42.6%) were positive by PCR assay. There were two major findings. First, the prevalence of *T. vaginalis* infection is indeed high. Although this could be due to our select patient group, it is also an indication of the existence of the infection in the study region. This high prevalence is probably due to the fact that, the *T. vaginalis* problem has long existed in our community, and the lack of awareness and routine laboratory detection may have compounded the problem. Second, the results of the present study point to *T. vaginalis* as being prevalent among even males with no genitourinary symptoms and not just females. It is very difficult to predict the *T. vaginalis* infection without clinical findings, as in our study only 33% (*n* = 10/30) and none (*n* = 0/39) respectively of the male and female patients with no genitourinary findings were positive for *T. vaginalis*. Overall, *T. vaginalis* positivity was high (48.3%) among female. The female gender also constituted significant risk factor for *T. vaginalis* with increased infection odds of 24.9 (Table 5). The prevalence obtained for females in this study is slightly above the WHO estimate (18.12%) in the African region [1] although high sensitive detection methods were included in the latter. The prevalence of *T. vaginalis* has generally been considered to be low among men, with a global prevalence of 1–5% [1, 24, 25]. The variation in prevalence observed between males and females is attributed to biological differences, particularly oestrogen, between gender. Also availability of iron [26] may facilitate persistent *T. vaginalis* infection among females during reproductive years.

In communities with prevalent *T. vaginalis* infections, an ideal detection test must have the greatest ability to pick true positives without selecting false negatives. Our results concerning *T. vaginalis* diagnosis demonstrated poor agreement between PCR assay as gold standard and the wet mount microscopy or JD’s *Trichomonas* V® test. Two observations merit attention. First, both wet mount microscopy and JD’s *Trichomonas* V® test tests recorded low sensitivities albeit with moderate to high specificities. It is noteworthy that, a certain proportion of false positives is often acceptable if the sensitivity margins are signicantly high. Yet, our wet mount microscopy could only detect 25 of 64 *T. vaginalis* positives and the rapid tests, 28 of 64 *T. vaginalis* positives. The negative likelihood ratios for both tests ranged between 0.1 through 0.52 depending on the patient population. Such low detection rates are more apt to frequent misdiagnosis and underestimation of the *T. vaginalis* problem. Second, in the search for less invasive human samples for detection of *T. vaginalis*, researchers have evaluated the possible use of urine specimens [14, 27]. In this current study, we found no discrimination in PCR test results between vaginal/urethral swabs and urine samples. Rather, extremely poor diagnostic outcomes were observed for wet mount microscopy and JD’s *Trichomonas* V® test performed with urine samples as opposed to vaginal/urethral swabs. The addition of urine did not significantly improve the *T. vaginalis* detection.

It has been emphasized that routine implementation of the PCR test should be used for *T. vaginalis* diagnosis. But the detection of *T. vaginalis* by PCR in microbiology laboratories remains a contentious issue with some experts questioning its clinical relevance in view of the financial demands involved. More so the strategy involves the use of skilled expertise. In contrast, expert microscopy is abundant sub-Saharan Africa and easily adapted in microbiology laboratories for diverse routine work. It remains the conventional detection method for routine diagnosis of parasites in STI across many sub-Saharan African countries. The results however suggest that wet mount microscopy may have low ability in our subset population to discriminate between those individuals with the disease and without the disease. The finding agrees well with submissions by other researchers [14, 28]. The JD’s *Trichomonas* V® test did not appear to be a better alternative either. Suffice to say that the poor performance of the rapid antigen test contrasts the higher sensitivity (100%) reported elsewhere [29] but the basis if this discrepancy is unknown.

The spectrum of selected patient population affects the prevalence of studies. In multivariate logistic analysis, we noted knowledge of STI to be protective against *T. vaginalis* and that this pattern was associated with increasing age and is prevalent among the female gender. Nearly a third of participants had no knowledge of STI. Perhaps the high occurrence of *T. vaginalis* could be attributed to lack of awareness and ignorance of the public health implication of the infection. This observation should however be interpreted with the awareness that a greater proportion of females than males consented to participate in the study. This may have overemphasized the *T. vaginalis* burden.

## Conclusion

In conclusion, the sensitivity of wet mount microscopy was low, thus it is unreliable for Trichomoniasis screening among patients in our region. However, given that a negative wet mount does not rule out the infection with *T. vaginalis,* we suggest a mandatory PCR assay as gold standard laboratory method for confirmation of negative results. The JD’s *Trichomonas* V® test should not be considered as an alternative test.

## Abbreviations

CI: Confidence Interval
HVS: High vaginal swab
PCR: Polymerase chain reaction
PID: Pelvic inflammatory disease
STI: Sexually transmitted disease
T. vaginalis: *Tricchomonas vaginalis*
U/S: Urethra swab
UTI: *Urinary tract disease*
